# The More I Get to Know You, the More I Distrust You? Non-linear Relationship between Social Skills and Social Behavior

**DOI:** 10.3389/fpsyt.2016.00049

**Published:** 2016-03-29

**Authors:** Pablo Billeke

**Affiliations:** ^1^División de Neurociencia, Centro de Investigación en Complejidad Social, Facultad de Gobierno, Universidad del Desarrollo, Santiago, Chile

**Keywords:** social cognition, trustworthiness, theory of mind, schizophrenia, social interactions

In recent years, there has been growing interest in the study of social skills in neuropsychiatric diseases (1, 2). This interest arises from the evidence, which indicates that social skill impairments generate deep impact on the functioning and, ultimately, the quality of life of neuropsychiatric patients. The better understating of the mechanisms involved in these processes is useful for elaborating therapeutic interventions to integrate these patients into society. However, the way in which the alteration of social skills specifically biases social behavior is not clear yet (3). In this context, the study carried out by Prevost et al. (4) may shed light in this important, yet unexplored, area. Here, the Reading the Mind in the Eyes test stimuli are used to measure both theory of mind (ToM) skills and the judgment of trustworthiness in strangers. The authors show that ToM affects the way in which healthy subjects trust strangers. Although prior evidence indicates that perspective-taking and empathy skills are positively related to trustworthy judgments, the authors find a negative relationship between these judgments and ToM. In other words, people who read the intentions of others better tend to distrust strangers. These results can be useful to understand the relationship between classical social skills, for example, those tested in clinical settings and real social behavior. Now on the one hand, during ontogenic development, trust behaviors increase in relation to both ToM skills and the maturation of ToM-related brain networks (5, 6). On the other hand, studies using interactive social tasks apparently show opposite results. For example, people with high ToM scoring tend to make more unfair decisions and have more strategic (Machiavellian) behaviors (7). A possible scope for interpreting these results is to distinguish the capacity of reading social information from the way of using this information to adapt social behaviors. For example, adolescents with better perspective-taking ability not only trust in others but also have the will to punish others’ distrusting behaviors (8). Likewise, during the development of social skills, adolescents no longer have trust behaviors when they face unfair partners (5). Hence, the social information that subjects extract from other people can be used to carry out different social behaviors (e.g., cooperative or non-cooperative behaviors) depending on other factors, as for example, the context, prior history of interaction with the same person, etc.

In order to further understand the relationship between trust and ToM, Prevost et al. also analyze a group of patients with paranoid schizophrenia, which is known to have social interaction impairments (1, 3). Interestingly, they found that patients with schizophrenia demonstrated a positive relationship between ToM skills and trustworthy judgments as opposed to the negative one of the healthy subjects. Patients who have better ToM skills judge strangers as more trustworthy. In interactive social games, patients with schizophrenia tend to trust less in other people as well as to not modify their behaviors according to their partners’ behaviors (9). Additionally, in repeated strategic social interactions, patients demonstrate opposite patterns of interaction when facing human partners as compared to the healthy people (10). These evidences indicate that alterations in the intention attribution to others bias, in a specific way, the social behaviors of these patients. Notably, these behavioral patterns seem to be related to the activity of the temporopareital junction (10, 11), a key area related to ToM.

Interestingly, when Prevost et al. evaluated all subjects as one group, an inverted U-shaped relationship emerged. This kind of relationship may be indicative of the existence of two interrelated processes. Thus, integrating behavioral and neurobiological evidences, it is possible to postulate that there are at least two processes that influence social behavior in independent directions. Trustworthy judgment may be understood as a (hypothetical) social decision (i.e., trust or not trust). Therefore, following recent evidence in strategic social decision making, it is possible to claim that social decisions are influenced by both social guide processes and integrative (social and non-social) processes (12). In our case, the former is the way in which subjects extract social information from the environment or, in other words, the others’ intentions. The latter is how subjects use the preceding social information and integrate it with non-social information (e.g., personal preferences) in order to make adaptive decisions. Beyond the role of both the temporoparietal junction and the medial prefrontal cortex in classical ToM tasks, recent evidence relates these areas to the evaluation of others’ preferences during social interactions (13–15). In this process, people with schizophrenia demonstrate impairments that may lead to the positive relationship between ToM and trust. Finally, the integration of the social information with personal preferences has been related to ventromedial prefrontal cortex activity (13, 14). This brain region shows functional connections with both temporoparietal junction and dorsolateral prefrontal cortex (16, 17). This connectivity seems to be necessary when people face difficult social dilemmas, in which the compliance of social norms clash with the personal interest (16, 17). In other words, ventromedial prefrontal cortex integrates social and non-social information when complex strategies are required to implement (12). Hence, in healthy subjects, the negative relationship between ToM and trust may be understood as the implementation of complex social strategies. Since this is a poorly explored area of research, it is necessary to carry out further studies that could unravel the mechanisms by which impairments in specific social skills bias social performance in neuropsychiatric diseases (1, 18).

## Author Contributions

The author confirms being the sole contributor of this work and approved it for publication.

## Conflict of Interest Statement

The author declares that the research was conducted in the absence of any commercial or financial relationships that could be construed as a potential conflict of interest.
